# Use of intraoperative ultrasound in resection of early-stage borderline ovarian tumors: *A case series*

**DOI:** 10.1016/j.gore.2025.101817

**Published:** 2025-07-28

**Authors:** Karthiga Natarajan, Marilyn Boo, Samir A. Saidi

**Affiliations:** a School of Medicine, The University of Sydney, Australia; b Chris O’Brien Lifehouse, Australia

## Abstract

Borderline ovarian tumors (BOTs) commonly occur in women of reproductive age. For patients desiring fertility preservation, cystectomies are recommended as healthy ovarian tissue is spared. However, intraoperative identification of small BOTs can be challenging. Intraoperative ultrasound is a novel technique that enhances the visualization of anatomical structures, potentially allowing for a more precise excision of the ovarian lesion, which maximizes the preservation of healthy ovarian tissue. Although it has clear advantages, the literature on the use of intraoperative ultrasound in gynecological surgeries remains limited.

Cases: We describe a case series of four women aged between 20 and 33 years old who underwent ultrasound-guided laparoscopic cystectomy for early-stage BOTs.

Discussion: We demonstrate that intraoperative ultrasound in laparoscopic resection of early-stage BOTs improves visualization of the ovarian tissue, thereby minimizing the amount of healthy ovarian tissue excised, which optimizes fertility preservation in patients.

## Highlights

• Borderline ovarian tumors typically present as early lesions in women of reproductive age.
• Visualizing early lesions laparoscopically can be challenging when performing cystectomies.
• We demonstrate that the use of intraoperative ultrasound for laparoscopic resection of early-stage ovarian tumors is safe and feasible.

## 1 Introduction

Borderline ovarian tumors (BOTs) are epithelial tumors of low malignant potential that often present as early-stage lesions in women of reproductive age (Fischerova et al., 2012, Skírnisdóttir et al., 2008). They account for 14% to 15% of all primary epithelial ovarian neoplasms (Bjørge et al., 1997, Skírnisdóttir et al., 2008). BOTs are prognostically favorable when compared to epithelial ovarian carcinomas, attributable to their earlier stage at diagnosis, slower growth rates, and better surgical outcomes (Fischerova et al., 2012, Link et al., 1996). For patients wishing to preserve fertility, fertility-sparing surgical (FSS) interventions such as ovarian cystectomy or unilateral salpingo-oophorectomy are recommended over radical interventions like bilateral salpingo-oophorectomy.

Conservative approaches, while preserving fertility, are associated with a higher risk of disease recurrence when compared to radical interventions. A French multicenter study reports rates of recurrence following conservative management as ranging between 11% and 30.3%, whereas radical management carries a substantially lower reported risk of 0% to 1.7% (Poncelet et al., 2006). However, despite the risk of recurrence, current evidence suggests that most recurrences are non-malignant (Fischerova et al., 2012, Della Corte et al., 2022), and ultrasound has demonstrated potential in detecting early recurrences during routine surveillance (Fischerova et al., 2012). As such, FSS procedures remain safe and appropriate for early-stage lesions without any impact on overall survival (Fischerova et al., 2012, Kasaven et al., 2022). Therefore, in the context of early-stage BOTs, it is imperative that patients’ reproductive goals are prioritized alongside oncologic safety, as FSS procedures offer comparable prognostic outcomes to non-FSS procedures with appropriate surveillance.

Intraoperative ultrasound-guided laparoscopic resection is a novel technique that is being explored in the conservative management of BOTs (Jones et al., 2017). The use of intraoperative ultrasound allows for better visualization of the abnormal ovarian lesion to facilitate its excision while sparing healthy ovarian tissue. Visualizing an abnormal ovarian lesion and distinguishing it from healthy ovarian tissue can be challenging with laparoscopy, particularly if the BOT is small (<3 cm). The aim of this study is to demonstrate the application of intraoperative ultrasound for identifying ovarian lesions prior to cystectomy as a fertility-preserving approach in patients with early-stage BOTs.

## 2 Methods and materials

This study was conducted in the Gynecological Oncology department of Chris O’Brien Lifehouse between July 2020 and May 2024. During this period, 4 patients underwent intraoperative ultrasound-guided laparoscopic resection of their BOTs, localized to the ovary.

**Fig. 1:**
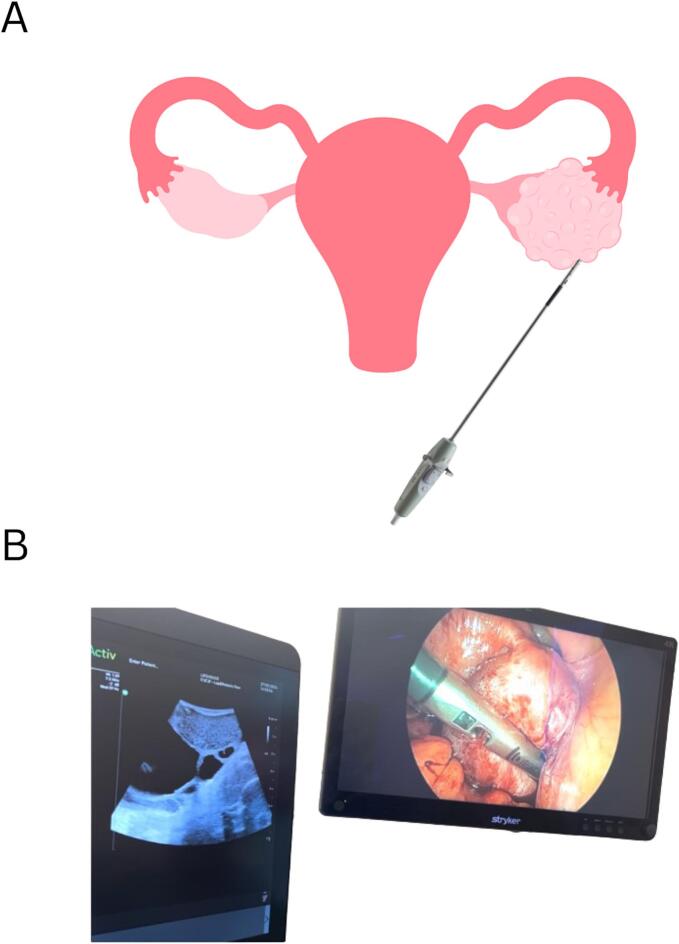
(A) The use of intraoperative ultrasound allows for the probe to be placed directly on the ovary, generating high-resolution images. (B) Real-time imaging with intraoperative ultrasound-guided resection of a serous epithelial borderline ovarian tumor (endometriosis is present).

Intraoperative ultrasound is a useful adjunct imaging tool that generates real-time high-resolution images during surgery. The transducer is placed directly on the organ of interest, providing images that are not impacted by overlying structures such as air, bone, or soft tissue (Lubner et al., 2021). (Fig. 1).

Clinical data from the 4 patients were collected for this study. This included information such as age, reproductive history, clinical symptoms, tumor markers, imaging findings, surgical methods, tumor histopathology, postoperative course, and relevant findings from the follow-up period. All the cases were pathologically diagnosed as serous epithelial borderline tumors following excision. The average age of these patients was 27.25 years (range, 20 to 33 years).

## 3 Cases

### 3.1 Case 1

A 27-year-old nulliparous female with a previous stage 2B serous borderline ovarian tumor (sBOT) was referred for the management of a complex ovarian cyst identified on serial ultrasound surveillance. She had previously undergone a laparoscopic right ovarian cystectomy.

**Fig. 2:**
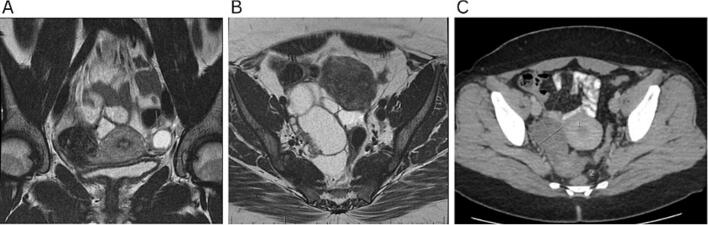
(A) Representative magnetic resonance axial image, T2-weighted, of left ovarian cyst measuring 20 mm (case 1). (B) Representative magnetic resonance axial image, T2-weighted, of right ovarian cyst measuring 96 x 50 x 56 mm (case 3). (C) Representative magnetic resonance axial image, T2-weighted, of right ovarian cyst measuring 16 mm (case 4).

On ultrasound surveillance, she was noted to have a persistent loculated left ovarian cyst that grew from 16 mm to 20 mm over 22 months from her initial surgery (Fig. 2A). Following multidisciplinary discussion, she underwent a laparoscopic left ovarian cystectomy with the use of intraoperative ultrasound. This approach allowed better differentiation of the abnormal cysts from her ovarian follicles, allowing two ovarian cysts to be surgically excised while conserving normal ovarian tissue. The patient had an uncomplicated postoperative recovery. She was able to undergo three cycles of oocyte cryopreservation following the second surgery, and no further persistent lesions requiring intervention were noted on ultrasound surveillance since the cystectomy.

### 3.2 Case 2

A 20-year-old nulliparous female was referred for the management of an ovarian cyst identified on an ultrasound for investigation of pelvic pain and dysmenorrhea. The physical exam was unremarkable. Investigations revealed a normal CA-125 level of 12kU/L with a right ovarian unilocular cystic lesion measuring 24 x 23 x 22 mm, with a solid component measuring 4 x 3 x 3 mm on ultrasound. The lesion was monitored closely, and a progressive increase in the size of the lesion to 24 x 23 x 21 mm with the solid component measuring 13 x 9 x 8 mm was noted over a period of 18 months. Following multidisciplinary discussion, a laparoscopic right ovarian cystectomy using intraoperative ultrasound was performed. Due to the small size of the cyst, this approach was chosen for more specific identification of the lesion, as it was not visualized macroscopically. The post-operative course was uncomplicated. The lesion was determined to be an sBOT on histopathology. No further persistent lesions requiring intervention were noted on ultrasound surveillance since then.

### 3.3 Case 3

A 33-year-old nulliparous female with a previous stage 3A sBOT was referred for the management of a complex ovarian cyst identified on serial ultrasound surveillance 3 months after her initial surgery. She had previously undergone a robotic left salpingo-oophorectomy and omental biopsy. This cyst was monitored closely with ultrasound surveillance over 25 months, after which she was lost to follow-up.

She presented again approximately 39 months later with pelvic pain and rectal discomfort. An MRI showed a 96 x 50 x 56 mm lesion comprised of multiple locules of variable sizes with solid enhancing internal tissue, suspicious for recurrence of a borderline tumor of the right ovary (Fig. 2B). There was minimal normal ovarian stroma seen. She was referred to a fertility specialist prior to her operation. Her AMH levels were found to be less than 1.0 ng/mL, indicating low ovarian reserve. Though fertility options were limited for this patient, as the MRI showed minimal remaining normal ovarian stroma, the patient declined elective oophorectomy.

A laparoscopic right ovarian cystectomy was performed 2 months after her re-presentation. Intraoperative ultrasound was utilized, which identified a small area of normal ovarian stroma for preservation. Histopathology confirmed an sBOT recurrence of the right ovary. The postoperative course was uncomplicated.

### 3.4 Case 4

A 29-year-old woman with a previous stage 2B sBOT was found to have a small lesion in the right ovary approximately 53 months after her initial laparoscopic left salpingo-oophorectomy and right cystectomy. This 12 mm complex lesion was monitored closely over 13 months. This cyst was noted to be increasing in size over this period, prompting further investigations.

On further investigations, MRI confirmed a right complex ovarian cyst measuring 16 mm, with mildly enhancing septations (Fig. 2C). Following multidisciplinary discussion, an ovarian cystectomy with intraoperative ultrasound was performed a month later to preserve her fertility. The patient was able to successfully conceive through IVF using her own oocytes following the surgery. She had no further persistent lesions requiring intervention since the cystectomy. (see Table 1 and Fig. 2).

**Table 1:** Case history and findings.

Case no.	Age at initial presentation	BMI	Para	Gynecological history	Radiology findings	Surgery	Final histopathology	Fertility outcomes
1	27	21	0	Stage 2B serous epithelial borderline ovarian tumor Previous laparoscopic right ovarian cystectomy with peritoneal excision and omental biopsies performed	20 mm left ovarian cyst containing a small nodule	Laparoscopic ultrasound-guided left ovarian cystectomy	Recurrent serous epithelial borderline ovarian tumor of the left ovary	Patient was able to undergo three cycles of oocyte cryopreservation following the second surgery

2	20	−	0	Nil	Cystic lesion measuring 24 x 23 x 21 mm with a solid component of 13 x 9 x 8 mm	Laparoscopic ultrasound-guided right ovarian cystectomy	Stage 1a serous epithelial borderline tumor of the right ovary	Nil

3	33	37	0	Stage 1A serous epithelial borderline tumor of the left ovary Previous robotic left salpingo-oophorectomy and omental biopsy performed	96 x 50 x 56 mm lesion comprised of multiple locules of variable sizes with solid enhancing internal tissue	Laparoscopic ultrasound-guided right ovarian cystectomy	Recurrent serous epithelial borderline tumor of the right ovary	Nil

4	29	27	0	Stage 2B serous epithelial borderline tumors Previous laparoscopic left salpingo-oophorectomy and partial right oophorectomy	16 mm complex cyst with mildly enhancing septations.	Laparoscopic ultrasound-guided right ovarian cystectomy	Recurrent serous epithelial borderline tumors of the right ovary	Patient conceived two children following surgery

## 4 Discussion

Ultrasound has been shown to detect small recurrences of BOTs during surveillance, potentially facilitating timely fertility-preserving interventions (Fischerova et al., 2012). The hypothesis is that the early excision of ovarian cysts preserves more normal ovarian tissue, thereby enhancing fertility outcomes. In certain cases, lesions detected during surveillance are small and challenging to accurately characterize intraoperatively. In the absence of intraoperative ultrasound to delineate normal ovarian tissue, cystectomy often results in the unintentional removal of healthy ovarian tissue. As BOTs typically affect young women, usually desiring fertility preservation and avoidance of iatrogenic menopause, surgical techniques should be optimized to maximize fertility preservation. In the management of early-stage disease where the lesion is localized and amenable to conservative excision, FSS procedures remain the mainstay of treatment in patients of childbearing age (Maramai et al., 2020). Therefore, surgical intervention should be performed as soon as possible to preserve the maximal amount of normal ovarian tissue. There is currently no specific size cutoff to determine when surgical intervention is indicated (Ekerhovd et al., 2001).

We demonstrate that the use of intraoperative ultrasound during laparoscopic resection is a safe, feasible, and novel approach for resection of early BOTs. When an early borderline tumor is seen, the options for treatment currently include removal of the whole ovary or expectant management until the cystic lesion is large enough to allow visualization and removal at surgery. The use of intraoperative ultrasound facilitates the removal of BOTs at a smaller size and therefore has the potential to minimize the volume of ovarian tissue removed.

A review by Crinti and Lin corroborates the use of intraoperative ultrasound as a novel yet reliable resource for gynecological surgeries, as it improves intraoperative visualization of anatomy and results in good surgical outcomes (Criniti and Lin, 2005). A retrospective study by Badiglian-Filho et al. reports that the use of intraoperative ultrasound facilitates conservative surgical management of benign tumors (Badiglian-Filho et al., 2019). Similarly, a prospective analysis by Jones et al. also reports the feasibility of safely conducting a fertility-sparing intraoperative ultrasound-guided ovarian wedge resection for small BOTs that may be hard to visualize laparoscopically (Jones et al., 2017).

Literature surrounding the use of intraoperative ultrasound in gynecological surgeries, particularly for ovarian cystectomies, is limited as it is a novel technique. Through this case series, we were able to demonstrate that the use of intraoperative ultrasound in the excision of BOTs allows for safe surgical outcomes while maximizing fertility preservation, adding to the existing literature.

Although BOTs have a favorable prognosis, especially when identified in the early stages, they are not without oncological risk. A minority of patients may present with an aggressive form that can negatively impact survival. Tropé et al. report that the 5-year survival rate for women with stage I borderline tumors is between 95% and 97% and between 65% and 87% for stage II-III tumors (Tropé et al., 2009). Malignant transformation was also noted in 20% to 30% of recurrent BOTs (du Bois et al., 2009). Therefore, clinical scenarios in which FSS procedures are advised against should be carefully considered. These procedures are often not recommended in patients with advanced-stage disease presenting with invasive peritoneal implants (Della Corte et al., 2022). It is also discouraged in sBOTs with micropapillary architecture, given their association with aggressive disease progression (Fischerova et al., 2012). In addition, more definitive procedures such as salpingo-oophorectomy are preferred when the tumor has progressed leaving little discernible healthy ovarian tissue or when clear surgical margins cannot be confidently determined (Fischerova et al., 2012, Della Corte et al., 2022). These findings highlight the importance of carefully considering reproductive goals with oncologic safety when making the clinical decision for FSS procedures.

## 5 Conclusion

Borderline ovarian tumors (BOTs) are considered benign lesions with low malignant potential that are prognostically favorable. BOTs predominantly affect women of reproductive age, for whom fertility preservation is a priority, making radical interventions less desirable in this population. The use of intraoperative ultrasound to guide laparoscopic ovarian cystectomy enhances the visualization of ovarian anatomy, particularly when the tumor is of a small diameter. This has the potential to optimize the preservation of healthy ovarian tissue, particularly in women with recurrent BOTs.

## Author contributions

All authors contributed to the preparation of this manuscript. KN performed a literature review, prepared the figures, and drafted the manuscript. MB provided supervision, clinical advice, and critical review. SAS conceptualized and designed the case series, provided supervision, and editorial revisions. All authors read and approved the final manuscript.

## Funding sources

No funding was received for this paper.

## Data Availability Statement

No data was generated.

## Consent Statement

Written informed consent was obtained from the patient for publication of this case report and accompanying images.

## CRediT authorship contribution statement

**Karthiga Natarajan:** Writing – review & editing, Writing – original draft, Visualization, Data curation. **Marilyn Boo:** Writing – review & editing, Validation, Supervision, Data curation. **Samir A. Saidi:** Writing – review & editing, Supervision, Methodology, Conceptualization.

## Declaration of competing interest

The authors declare that they have no known competing financial interests or personal relationships that could have appeared to influence the work reported in this paper.
